# Nutritional Status of Adult Patients with Pulmonary Tuberculosis in Rural Central India and Its Association with Mortality

**DOI:** 10.1371/journal.pone.0077979

**Published:** 2013-10-24

**Authors:** Anurag Bhargava, Madhuri Chatterjee, Yogesh Jain, Biswaroop Chatterjee, Anju Kataria, Madhavi Bhargava, Raman Kataria, Ravi D’Souza, Rachna Jain, Andrea Benedetti, Madhukar Pai, Dick Menzies

**Affiliations:** 1 Department of Internal Medicine, Himalayan Institute of Medical Sciences, Jolly Grant, Uttarakhand, India; 2 Krishak Maitri Hospital, Chengail, West Bengal, India; 3 Jan Swasthya Sahyog, Village and P.O.Ganiyari, Chhattisgarh, India; 4 Department of Microbiology, Himalayan Institute of Medical Sciences, Jolly Grant, Uttarakhand, India; 5 Department of Community Medicine, Himalayan Institute of Medical Sciences, Jolly Grant, Uttarakhand, India; 6 Centre for Public Health and Equity, Bhopal, Madhya Pradesh, India; 7 Respiratory Epidemiology & Clinical Research Unit, Montreal Chest Institute, McGill University Health Centre, Montreal, Quebec, Canada; 8 Department of Epidemiology, Biostatistics and Occupational Health, McGill University, Montreal, Quebec, Canada; University of California, United States of America

## Abstract

Under-nutrition is a known risk factor for TB and can adversely affect treatment outcomes. However, data from India are sparse, despite the high burden of TB as well as malnutrition in India. We assessed the nutritional status at the time of diagnosis and completion of therapy, and its association with deaths during TB treatment, in a consecutive cohort of 1695 adult patients with pulmonary tuberculosis in rural India during 2004 - 2009.Multivariable logistic regression was used to obtain adjusted estimates of the association of nutritional status with deaths during treatment. At the time of diagnosis, median BMI and body weights were 16.0 kg/m^2^and 42.1 kg in men, and 15.0 kg/m^2^and 34.1 kg in women, indicating that 80% of women and 67% of men had moderate to severe under-nutrition (BMI<17.0 kg/m^2^). Fifty two percent of the patients (57% of men and 48% of women) had stunting indicating chronic under-nutrition. Half of women and one third of men remained moderately to severely underweight at the end of treatment. 60 deaths occurred in 1179 patients (5%) in whom treatment was initiated. Severe under-nutrition at diagnosis was associated with a 2 fold higher risk of death. Overall, a majority of patients had evidence of chronic severe under-nutrition at diagnosis, which persisted even after successful treatment in a significant proportion of them. These findings suggest the need for nutritional support during treatment of pulmonary TB in this rural population.

## Introduction

India has a major share of the global incidence of tuberculosis (TB) (including MDR-TB) and TB related mortality. According to estimates for 2010, TB incidence was 185 per 100,000 with 320,000 TB-related deaths[1]. HIV infection plays a relatively minor role in the TB epidemic in India[2], whereas under-nutrition, smoking, and diabetes are considered more important risk factors[3–5]. Recent systematic reviews have established the role of under-nutrition as an important risk factor for reactivation of latent TB infection to TB disease [6,7], while obesity has been found to be protective against TB[8–10]. Under-nutrition is the most widely prevalent risk factor, accounting for the highest population attributable risk (PAR) for TB in India[11]. In India more than one third of women and men in the age group of 15-49 years are under-nourished(BMI <18.5 kg/m^2^), and nearly half of children under the age of five years have moderate to severe under-nutrition(as defined in WHO child growth standards)[12].

Tuberculosis can lead to or worsen pre-existing under-nutrition, by decreasing appetite, and by increased catabolism [13,14].High prevalence of under-nutrition in TB patients has been reported in other settings[15–19],and has been linked to excess deaths[16],and increased risk of relapse[20].There have been few reports on prevalence, severity and implications of under-nutrition in Indian patients with TB [21],particularly from India’s rural areas, where the majority of Indians live, and which have a higher prevalence of poverty, under-nutrition and self-reported TB than urban areas[12]. We assessed nutritional status and selected treatment outcomes in a consecutive cohort of 1695 patients over 18 years of age diagnosed with pulmonary tuberculosis at a rural hospital and community health programme in central India between 2004-2009.

## Materials and Methods

### Study subjects

Chhattisgarh state, a state in central India, has a population of 20 million of which more than a third is of indigenous people - a marginalised social group, with higher rates of poverty, illiteracy, infant and maternal mortality and under-nutrition, than the average Indian population[12,22,23]. Seventy-two percent of the population lives in poverty, defined by the multi-dimensional poverty index[24,25].The economy is based on cultivation of rain-fed rice crops; many residents migrate seasonally to other states for livelihood . Our study was conducted at Jan Swasthya Sahyog (JSS-or People’s Health Support Group), a non-profit voluntary organisation that has provided health services in Bilaspur district of Chhattisgarh state since 2000. JSS caters to the rural population through its community health programme in 54 villages, a secondary care level hospital which is accessed by people of over 1500 villages, and 3 outreach clinics serving the villages in the community health programme and other remote villages. All patients with pulmonary tuberculosis 18 years of age and older, diagnosed at the hospital, and the 3 outreach clinic centres between January 1, 2004 and 31 December, 2009, were included in the study. Patients were diagnosed primarily on the basis of sputum smear microscopy and radiography, according to WHO guidelines[26]. Smear positive pulmonary tuberculosis was diagnosed if microscopic examination of one or more direct smears of sputum was positive for acid-fast bacilli on Ziehl-Neelsen staining. Smear negative pulmonary tuberculosis was diagnosed if a patient with symptoms suggestive of pulmonary TB had two smear examinations negative for acid-fast bacilli but had chest radiographic appearance judged by the physicians to be compatible with pulmonary TB[27]. Patients were categorised on the basis of treatment history as new cases or previously treated cases[26,27].

This study was a secondary analysis of routinely collected clinical data at JSS hospital and community health programme during the period 2004 - 2009. Analysis of the data without patient identifiers was done at McGill University after ethics approval from the Institutional Review Board at the Faculty of Medicine, McGill University, Montreal (IRB Study Number: A10-M111-11B). The protocol approved by the IRB noted that no informed consents were obtained because the data were collected as part of routine hospital care, and analysed post-hoc as secondary data.

### Study design

This retrospective cohort study was conducted with the following objectives: 

1. To assess the nutritional status of patients with active pulmonary TB, before and at the completion of therapy.

2. To assess the association of the pre-treatment nutritional status with mortality during TB treatment.

### Methods

Information on age, gender, residence, sputum smear status, grade of sputum smear, previous history of treatment, history of any illness or death in a family member diagnosed as TB, was recorded for each patient. Weights were recorded at the time of diagnosis and at completion of treatment using the same regularly calibrated beam balance (+100 g precision), with the patient wearing light clothing. Heights were recorded to the nearest centimeter with a stadiometer using standard procedures. Measurements were made by trained paramedics. 

In patients where both heights and weights were obtained, body mass index (BMI in kg/m^2^) was calculated and the patients were classified into categories based on the BMI cut-offs for weight categories as recommended by the WHO [28,29]. The Indian Council of Medical Research(ICMR) has recommended weights and heights of 60 kg and 172 cm for adult Indian men, and 55 kg and 162 cm for adult Indian women; these were used as reference weights and heights in this study[30]. The Indian reference standard for height was a single value and for purpose of comparison of the distribution of heights, the National Center for Health Statistics (NCHS) reference standards were used[31]. Stunting (Short stature)was defined as height below - 2 standard deviations from the median of the NCHS reference heights for 18 year old men and women[31], as has been done in some other studies on nutritional status from India[32]. We used the body weight as well as the BMI in our analyses. Use of body weights allowed comparisons with studies from the Revised National Tuberculosis Control Program (RNTCP), and older studies (which recorded only weight). Use of BMI enabled classification into WHO recommended weight categories[28,29].

Patients diagnosed with active TB were advised to seek free treatment under the RNTCP as a first option. Patients who opted for treatment at JSS received daily, self-administered, short course therapy prescribed according to WHO Guidelines[26]. Anti-TB drugs were provided at highly subsidised rates ($ 1 per month) or were free. Patients were counselled about TB, its treatment, cessation of smoking, and avoidance of alcohol use. Based on the location of their residence patients were classified into the following groups. Group 1: if the residence was related to the village health programme and outreach clinics. Group 2: if residence was within a 20 km radius of the hospital. Group 3: if residence was not related to the village health programme/outreach clinics and was beyond 20 km from the hospital.

Outcomes were classified according to WHO guidelines as ‘Cured’ or ‘Treatment completed’ (these categories together constituted “treatment success”) ‘Deaths’, ‘Defaults’, ‘Treatment failure’, or ‘Transferred out’(i.e. those who sought care under RNTCP)[26]. TB deaths refer to any deaths occurring during the course of TB treatment [26], and these were ascertained from hospital medical records, during home visits made to Group 1 and Group 2 patients, or communications from relatives in response to phone calls or postal reminders in Group 3 patients. 

### Statistical analysis

Association between categorical variables was assessed using the Fisher’s exact test. Student’s t tests (paired and unpaired), and Mann-Whitney tests were used to compare means, medians respectively as appropriate. The multivariable logistic regression analyses of outcomes of TB deaths and treatment success were performed with nutritional status (weight, BMI) as the predictor variables and were adjusted for demographic, clinical and disease related covariates. Variables were included in the final model if the univariate analysis showed a p value < 0.25, or if the variable was of known clinical importance[33]. Age, weight and BMI were included as continuous variables in the regression model after verifying the linearity of associations under study in a generalised additive model. Patients who defaulted were excluded in the analyses of death. Statistical analysis was done using STATA 11 (Stata Corp, College Station, TX).

## Results

The characteristics of patients are described in table 1. 1695 patients were diagnosed with pulmonary tuberculosis during 2004-2009. Of these patients, measurements of body weight and height at diagnosis were made in 1665 (98.2%) and 1523 (89.9%) respectively. 1179 patients of these 1695 patients were treated at JSS, while 516 patients were transferred out for treatment under the RNTCP. The patients transferred out for treatment under RNTCP included a slightly higher proportion of men (71.5% vs. 66.4%, p=0.04), who were older than the men who opted for treatment at JSS (median age of 45 years compared to 39 years in the JSS group, p <0.0005), and a higher proportion of patients from distant towns and villages. Those transferred out to the RNTCP were more likely to be smear positive (70% vs. 64 %. p=0.03). The patients treated at JSS included a higher proportion of women, patients with previously treated disease (19.6% vs.13.4%, p=0.002), and included all the patients with HIV associated pulmonary TB. There was no significant difference in the pre-treatment BMI and weights of men and women in the JSS treated group compared to the group of patients who were transferred out to the RNTCP. 

**Table 1:** Demographic, clinical, and anthropometric characteristics of all adult patients diagnosed with pulmonary tuberculosis at JSS (2004-2009).

**Characteristic**	**N=1695**	
**DEMOGRAPHIC AND GEOGRAPHIC**		
Median age in years, (IQR)	38 (29,50)	
Men	40 (30,52)	
Women	35 (27,43)	
Gender, (%)		
Men	1152 (68)	
Women	543 (32)(32)	
Residence, (%)		
Rural	1601 (94)	
Urban	94 (6)	
Location of residence, (%):		
Group1: related to village health program/outreach clinics	193 (11)	
Group 2: within a radius of 20 km of hospital	161 (9)	
Group 3: beyond 20 km radius of hospital	1341 (80)	
**DISEASE RELATED**		
Sputum smear, (%)		
Smear positive	1119 (66)	
Smear negative	576 (34)	
Sputum grade, (%)		
1+	357 (32)	
2+	302 (27)	
3+	460 (41)	
Treatment history, (%)		
New cases	1395 (82)	
Retreatment cases	300 (18)	
**CO-MORBIDITIES**		
No. of patients with HIV infection , % of all HIV tests during 2004-9	91 (3.2)	
No. of patients with Pulm. TB- HIV co-infection, % of all HIV tests in pulm. TB	39 (4.4)	
No. of patients with pulmonary TB & Diabetes mellitus,(%) of tested	77 (11.2)	
No. of patients with anemia (Hemoglobin.less than 12 g/dl), % of tested	515 (73.1)	
severe anemia (Hemoglobin.less than 8 g/dl), % of tested	143 (20.3)	
**FAMILY HISTORY OF TB**		
No. of patients with family history of TB, (%)	389 (23)	
No. of patients with family history of TB related death ,	207(12.2)	
**ANTHROPOMETRIC DATA:**		
**Weight at diagnosis**	**Men (n=1128)**	**Women (n=537)**
Weight in kg, Median(IQR)	42.0 (37.5, 47)	34.1 (30.4, 38.1)
Height* in cm, Median (IQR)	162 (158,165)	151 (148,154)
**Body mass index (BMI**)** at diagnosis**	**Men (n=1030)**	**Women (n=493)**
Body mass index (BMI)* in kg/m^2^, Median(IQR)	16.0 (14.6, 17.4)	15.0 (13.6,16.5)
BMI> 30 kg/m^2^: Obese, (%)	0 (0)	0 (0)
BMI 25.0-29.99: Overweight, (%)	2 (0.2)	1 (0.2)
BMI 18.5-24.99: Normal, (%)	130 (12.6)	32 (6.5)
BMI 17.0-18.49: Mild underweight, (%)	204 (19.8)	63 (12.8)
BMI 16.0-16.99: Moderate under underweight, (%)	179 (17.4)	70 (14.2)
BMI <16.0: Severe underweight, (%)	515 (50.0)	327 (66.3)

* Height measured in 1030 men and 493 women

**Figure 1:**
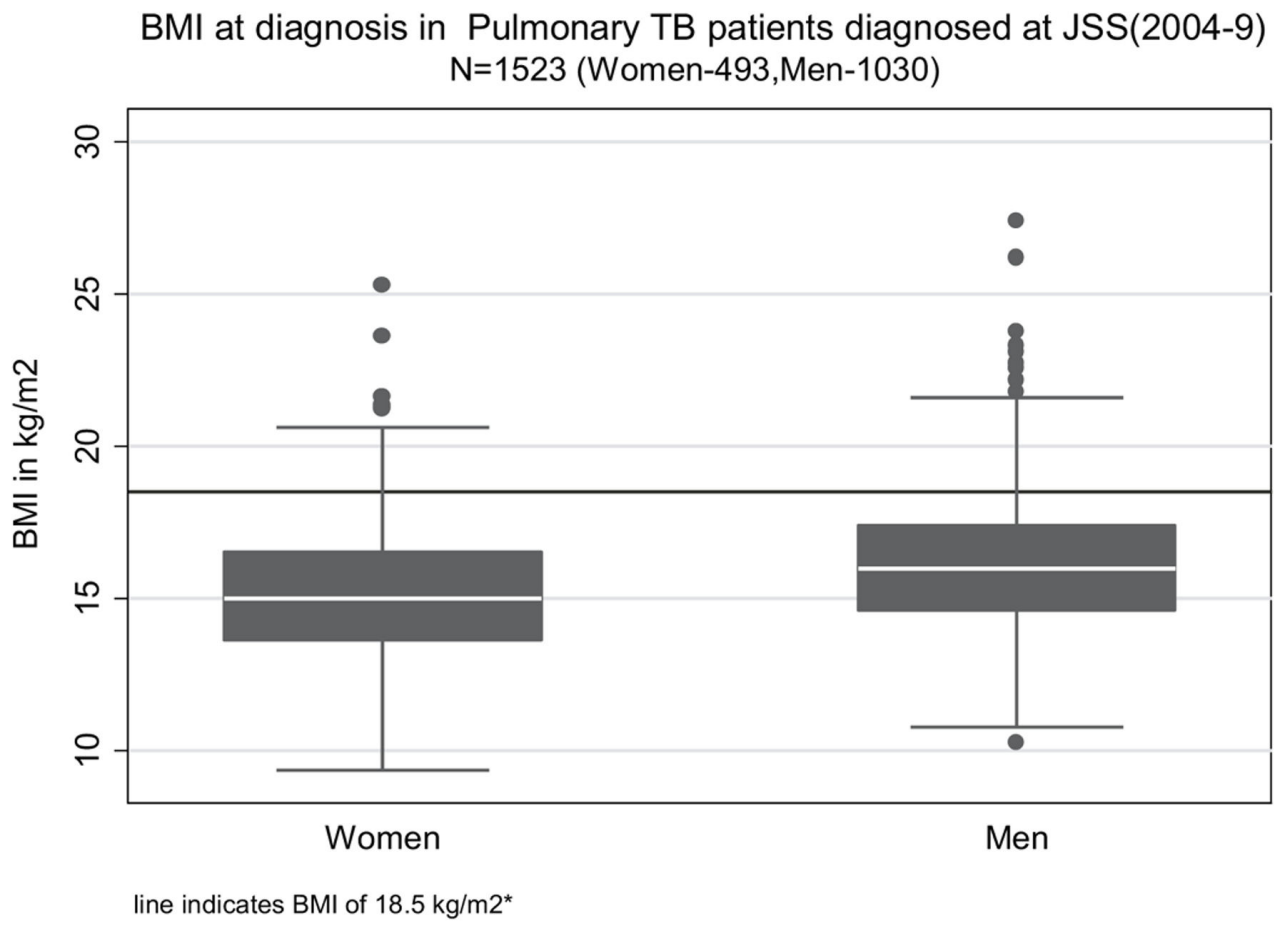
Distribution of body mass index (BMI) at diagnosis in adult patients with pulmonary Tuberculosis at JSS, 2004-9. *BMI of 18.5 kg/m^2^ is the cutoff separating under-nutrition (BMI<18.5 kg/m^2^) from normal range of BMI (18.5-24.99 kg/m^2^).

Out of the 1179 patients treated at JSS, 756 were treated successfully (64.1%), 60 patients died(5.1%), 4 patients failed treatment (0.3%), 354 (30%) patients interrupted treatment before completion (defaulted), and in 5 (0.4%) patients the outcomes were unknown. The majority of the subjects were men from rural areas, and had smear positive pulmonary TB. More than two-thirds of subjects with smear positive pulmonary TB had a high bacterial load with more than 1 acid fast bacillus seen per oil immersion field (sputum smear grade of 2+ and higher)on direct microscopy (table 1). 80% of patients came from a distance of more than 20 km from the hospital. A significant proportion had history of a family member with active TB; of these, many had died. After under-nutrition the most common co-morbidities were anemia, diabetes and HIV infection, although these were tested (with appropriate informed consent) in 705, 686, and 893 patients respectively (table 1).Table 1 also shows the anthropometric status of the patients at diagnosis, while Figure 1 shows the distribution of BMI of men and women patients at diagnosis .The median pre-treatment BMI in men and women was about 16 kg/m^2^ and 15 kg/m^2^ respectively, while the median weight was 42.0 kg and 34.1 kg in men and women respectively. More than 85% of men and almost 95 % of women were underweight at diagnosis (BMI <18.5 kg/m^2^), and two-thirds of women and half of men were severely underweight (BMI < 16 kg/m^2^) (table 1). 

Overall, 843 of 1523 (55%) of patients whose BMI was measured had severe underweight at diagnosis (data not in table). There were 72 men and 30 women with a BMI< 13 kg/m^2^ and BMI < 12 kg/m^2^ respectively (data not shown in table); the lowest value of BMI was 9.37 kg/m^2^.As seen in table 1, the pre-treatment weights were at least 30% lower, and the heights were 10 cm shorter than the Indian Council of Medical Research (ICMR) recommended reference measures [30]. Only 2.4% of patients had pre-treatment weights equal to or above the suggested Indian reference weights[30]. 52% of patients (57% of men and 48% of women) had stunting with heights below the 3^rd^ percentile of the National Center for Health Statistics(NCHS) standards for height at 18 years of age (163 cm for men and 151 cm for women). This poor attainment of adult stature suggests chronic inadequate nutrition in childhood during the growth period [34,35]. There was no statistically or clinically significant difference in *weights* of men and women according to smear status, treatment history, HIV and diabetes status, year of diagnosis or location of residence (data not shown in table).

The clinical, demographic and anthropometric indicators in 1179 patients treated at JSS stratified by outcomes are shown in table 2. Older age, male gender, residence more than 20 km from the hospital, & HIV status were associated with both death and default.The median (IQR) ages of death was 42 (35,55) years in men and 32.5 (27,50) years in women. Patients with a previous history of TB treatment had a higher rate of default. Men who died during treatment had significantly lower pre-treatment weights than those who were treated successfully (4 kg lower median weight), but in women this difference was not statistically significant. Overall, among patients treated at JSS, 71% were moderately to severely underweight at diagnosis, while in those who died this proportion was 80% (p=0.08, Fisher’s exact test-data not shown in table). There was no significant difference in baseline weights between men and women who completed treatment successfully compared to those who defaulted before completion. 

**Table 2:** Demographic, clinical and anthropometric characteristics of 1179* adult patients with pulmonary tuberculosis who were treated at JSS (2004-2009), stratified by outcomes**.

**Characteristic**	**Treatment success # N=756**	**TB death § N=60**	**Default ^//^ during TB treatment N=354**	**P values (Treatment success vs. TB death)**	**P values (Treatment success vs. default)**
**DEMOGRAPHIC & GEOGRAPHIC**					
Median age in years, (IQR)	35 (27,45)	40 (35,53)	40 (29,52)	P=0.0002^†^	P=0.0001^†^
Gender, (%)					
Men	479 (63)	46 (77)	251 (71)	P=0.05^‡^	P=0.02^‡^
Women	277 (37)	14 (23)	103 (29)		
Residence, (%)					
Rural	722 (96)	54 (90)	327 (92)	P=0.07^‡^	P=0.05^‡^
Urban	34 (4)	6 (10)	27 (8)		
Location of residence, (%)					
Group 1: related to village health program/outreach clinic	140 (18)	11(18)	34 (10)	P=0.27^‡^	P<0.0005^‡^
Group 2 <20 km from hospital	87 (12)	11 (18)	32 (9)		
Group 3: >20 km from hospital	529 (70)	38 (64)	288 (81)		
**DISEASE RELATED**					
Sputum smear, (%)					
Smear positive	499 (66)	37 (62)	215 (61)	P=0.48^‡^	P=0.09^‡^
Smear negative	257 (34)	23 (38)	139 (39)		
Sputum grade, (%)					
1+	156 (31)	10 (27)	60 (28)	P=0.16^‡^	P=0.31^‡^
2+	151 (30)	7 (19)	59 (27)		
3+	192 (39)	20 (46)	96 (45)		
Treatment history, (%)					
New cases	628 (83)	46 (77)	269 (76)	P=0.22^‡^	P=0.007^‡^
Retreatment cases	128 (17)	14 (23)	85 (24)		
**CO-MORBIDITIES**					
No. of patients with HIV co-infection, %	9 (23)	10 (26)	20 (51)	P<0.0005^‡^	P<0.0005^‡^
**ANTHROPOMETRIC DATA**					
Weight at diagnosis[Median (IQR)]					
Men	[n=478]42.5 (37.8,47.3)	[n=46]38.5 (34.4,44.6)	[n=245]41.7 (37.1,47.5)	P=0.002†	P=0.35†
Women	[n=277]34.3 (30.4,38.8)(30.4,38.8)(30.4,38.8)	[n=14]32.7 (29,38.1)	[n=100]33.9 (30,37.3)	P=0.46†	P=0.24†
BMI at diagnosis [median(IQR)]					
Men	[N=459]16.1 (14.7,17.5)	[N=46]15.0 (12.7,16.4)	[n=219]15.8 (14.4,17.4)	P=0.0002†	P=0.27†
Women	[N=269]15.1 (13.7,16.8)	[n=14]14.6 (12.8,17.8)	[N=83]14.7 (13.4,16)	P=0.65†	P=0.05†

*Excludes 5 patients with unknown outcomes & 4 patients with treatment failure. ** 516 patients who were “transferred out” to the RNTCP &did not have their treatment outcomes recorded have been excluded in this analysis. ‡ Fisher exact test. † Mann-Whitney test. # this includes patients who were cured and those who completed treatment. § Death due to any reason occurring during treatment of TB. // Interruption of therapy for 2 consecutive months or more.

**Figure 2:**
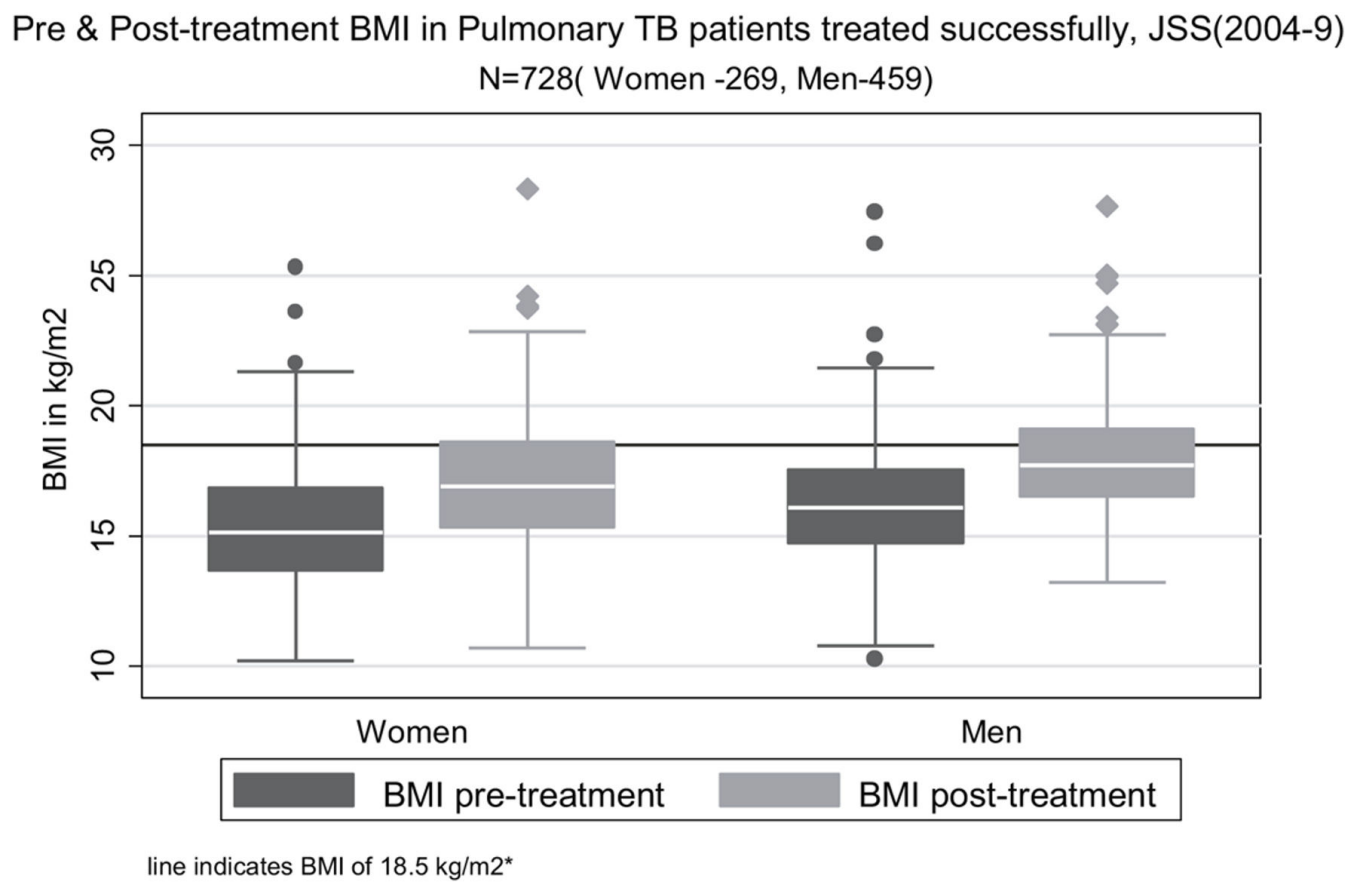
Distribution of pre-treatment and post-treatment body mass index (BMI) of adult patients with pulmonary tuberculosis treated successfully at JSS, 2004-2009. *BMI of 18.5 kg/m^2^ is the cutoff separating under-nutrition (BMI<18.5 kg/m^2^) from normal range of BMI (18.5-24.99 kg/m^2^).

Weights and BMIs of patients after successful treatment are shown in table 3. These weights were around 10% higher than pre-treatment weights, with an overall median weight gain of 3.9 kg (IQR: 1.9, 5.9) in both sexes. Only about one-third of men and a quarter of women had BMIs in the normal range after successful treatment (see Figure 2). Of the patients who completed treatment successfully, more than half of women and one-third of men remained moderately to severely underweight (BMI<17 kg/m^2^).

**Table 3:** Pre-treatment, post-treatment weights & body mass index (BMI) in 756 patients with pulmonary TB who completed treatment successfully, (2004-2009).

	**Pre-treatment**	**Post-treatment**
**Measurements**		
**I. Weights at diagnosis and post-treatment in kg, n=756**	(Men-479; Women-277)	(Men-479; Women-277)
a. Weight in kg, Median (IQR)		
men	42.7 (37.8, 47.3)	47.1 (42.3,51.2)
women	34.9 (30.5,38.8)	38.8 (34.3,42.6)
b. Weight change with treatment‡ , median (IQR)	3.9 (1.9,5.9)	
men	4.1 (2.0,6.0)	
women	3.7 (1.5,5.9)	
c. Weight change as a proportion of pre-treatment weight §		
men	9.3 (4.5,14.3)	
women	10.8 (3.8, 17.8)	
**II. Distribution of Body mass index (BMI**)** in kg/m^2^,**n=728	(Men-459; Women-269)	(Men-459; Women-269)
BMI> 30 : Obese, (%)		
men	0 (0)	0 (0)
women	0 (0)	0 (0)
BMI 25.0-29.99: Overweight, (%)		
men	2 (0.4)	2 (0.4)
women	1 (0.4)	1 (0.4)
BMI 18.5-24.99: Normal, (%)		
men	58 (12.6)	163 (35.6)
women	19 (7.1)	70 (26)
BMI 17.0-18.49: Mild underweight, (%)		
men	93 (20.2)	131 (28.5)
women	41 (15.2)	59 (21.9)
BMI 16.0-16.99: Moderate underweight, (%)		
men	89 (19.4)	85 (18.5)
women	40 (14.9)	45 (16.7)
BMI <16.0: Severe underweight, (%)		
men	217 (47.4)	78 (17.0)
women	168 (62.4)	94 (35.0)

^‡^ Weight change with treatment = [post-treatment weight – pre-treatment weight]

^§^ Weight change as a proportion of pre-treatment weight= [weight change/pre-treatment weight *100]

Age, pre-treatment weight, height, BMI, and gender were significantly associated with death during TB treatment (table 4). HIV positive status had the strongest association with TB related deaths (table 4). Death during treatment was significantly lower with greater pre-treatment body mass index, when adjusted for the covariates of age, gender, treatment category, sputum smear and HIV status. The adjusted OR (aOR) for each unit increase in BMI was 0.78(CI 0.68, 0.90). The aOR for TB death for men and women at their median levels of BMI (BMI 16.0 in men and 15.0 in women) compared to 18.5 kg/m^2^ (cut-off for normal BMI) were 1.9 (CI 1.3 to 2.6) for men, and 2.4 (CI 1.4 to 3.9), while the aOR for TB death at a BMI of 13 kg/m^2^ was 3.9 (CI 1.7 to 8.3). In a subgroup analysis the association of pre-treatment BMI with TB death was found to be statistically significant only in men. However, when we included an interaction term between BMI and sex, this interaction term was not significant (p=0.09). Gender was significantly associated with death during treatment in univariable analysis but was not associated with treatment outcomes when height was included in the model. 

**Table 4:** Crude and adjusted estimates of covariates associated with TB related mortality.

**Variable**	**Crude OR for TB death ( with 95% CI)**		**Adjusted OR§ for TB death ( with 95% CI)**	
	OR	95% CI	OR	95% CI
**Age**(per 10 years older)	**1.48** *	(1.10,1.97)	**1.34** *	(1.10,1.79)
**Gender**				
Women	1	(reference)	1	(reference)
Men	**1.90**	(1.03, 3.52)	1.59	(0.67, 3.75)
**Pre-treatment weight (per 5 kg greater)**	**0.82** **	(0.70,1.00)	**0.59** **	(0.47,0.77)
**Height at diagnosis (per cm taller)**	1.03	(0.99, 1.06)	**1.06**	(1.0, 1.12)
**Body mass index^‡^ (per unit increase)**	0.80	(0.71,0.91)	**0.78**	(0.68,0.90)
**Sputum status**				
Smear negative	1	(reference)	1	(reference)
Smear positive	0.83	(0.48, 1.42)	1.0	(0.55, 1.85)
**Sputum grade ^‡‡^**				
0 (smear negative)	1	(reference)	1	(reference)
1+	0.72	(0.33,1.5)	0.85	(0.37,1.94)
2+	0.52	(0.22,1.24)	0.67	(0.26,1.68)
3+	1.16	(0.62,2.18)	1.56	(0.78,3.10)
**HIV status**				
HIV negative	1	(reference)	1	(reference)
HIV positive	**16.6**	(6.45,42.7)	**19.78**	(6.83,57.31)
**Treatment category**				
New case	1	(reference)	1	(reference)
Previously treated case	1.49	(0.80,2.80)	1.24	(0.62,2.48)
Location of residence ***				
group 1:	1	(reference)	NA	NA
group 2:	1.61	(0.67,3.83)	NA	NA
group 3:	0.91	(0.46,1.83)	NA	NA
**Family history of TB**				
No history of TB	1	(reference)	NA	NA
History of TB +	1.14	(0.63,2.09)	NA	NA

^§^ Adjusted for age, gender, pre-treatment weight, height, sputum status, HIV status, treatment category.

^‡^ For logistic regression of TB death on BMI, weight, height were omitted from the model

^‡‡^ For logistic regression of TB death on sputum grade, sputum smear status was omitted from the model

* OR scaled to represent increase of age of 10 years.

** OR scaled to represent increase of weight of 5 kg.

*** Group 1: related to village health program/ outreach clinics. Group 2: within a 20 km radius of the hospital. Group 3: beyond a 20 km radius of the hospital. NA: Not applicable as variable is not in final mode**l**

## Discussion

In this study, under-nutrition was the most prevalent co-morbidity, present in more than 85% of rural men and women with pulmonary TB at diagnosis, of which more than two thirds were moderately to severely underweight according to BMI based criteria. Levels of BMI which are considered lethal [36]- BMI< 13 kg/m^2^ and BMI <11 kg/m^2^ in men and women respectively- were seen in 5% of patients. Women were more likely to have severe nutritional deficit. More than half of women and one third of men continued to be moderately to severely underweight at the end of successful treatment. The deaths which occurred during TB treatment were premature--defined as those occurring belowthe age of 65 years[37]. The median ages at death (42 years in men and 32 years in women) were in contrast to the current life expectancy of 65.8 years for men and 68.1 years for women in India[38]. A BMI of 16.0 kg/m^2^ was associated with a 2 fold higher odds of death. The association observed between BMI and death during TB treatment was mainly driven by a strong association in men.  This could have been due to a number of reasons. With 14 documented deaths in women, the study had low power to study subgroup effects. Another explanation can be the very high prevalence of severe under-nutrition in the women (more than 60%). At such high prevalence the predictive value of a risk factor may be lost. There could be a biologic reason, as women have greater ability to adapt to starvation, given that the level of BMI that is considered lethal in women ( 11 kg/m^2^) is lower than in men(13 kg/m^2^)[36]. 

The prevalence of severe under-nutrition in our cohort was higher than that reported in HIV positive patients in urban India and sub-Saharan Africa (table 5) [16,21,39]. In a report from Malawi,where the prevalence of HIV infection was 80% among TB patients, the prevalence of severe under-nutrition was 21% in contrast to nearly 50% in our cohort (table 5) [16] . The body weights were in fact close to those of poor urban patients of the famous Madras (now Chennai) trial 5 decades ago (table 5) [40]. In the RNTCP, where 1.5 million patients are diagnosed per year, body weights are recorded but have not been reported. The weight gain in this study was similar to the mean weight gain of 3.2 kg observed in another study in South India [41]. Low BMI has been reported as an independent risk factor for all cause and TB related mortality in population based cohorts [42–44]. In TB patients, under-nutrition has been associated with increased risk of TB related mortality, including those with drug resistant TB and HIV infection [9,16,45–51]. The strength of the association of under-nutrition with mortality in this study was broadly consistent with other studies.BMI of less than 17 was associated with a mortality rate ratio of 1.7 [46],compared to a 4 fold increased risk of death associated with weight < 35 kg in a cohort from south India [50].

**Table 5:** Comparative weights and body mass index in patients with pulmonary TB from studies in India and other high TB burden countries.

**Country ,Year, (reference)**	**Setting**,	**No. of patients**	**Percent HIV positivity**	**Weight (men**)**, mean**	**Weight (women), mean**	**BMI in men, mean**	**BMI in women, mean**
India (south), 1959[40]	Urban	193	0 (Pre-HIV era)	39.8	33.3	----	-----
India (south),2008[21]	Urban	174	100 %	50.2	43.2	18.7	18.7
India(central), 2004-2009[present study]	Rural	1695	2.3 %*	42.3	34.6	16.1	15.2
Malawi, 2002[16]	Rural	1181	80 %	---	---	18.4	17.9
Tanzania,2006[39]	Urban	2231	32 %	52.7	47.2	18.95	19.3
Indonesia, 2010[58]	Rural	300	Not tested	---	---	16.5 (both sexes)	

* calculated as number of patients with HIV-pulmonary TB co-infection / total number of patients with pulmonary TB during 2004-9.

 Our study has certain limitations. Heights were missing in about 10% of subjects, but the weights, demographic and clinical characteristics of subjects with missing values were similar to those without missing values. We could not comment on specific loss with regard to lean body mass or body fat or micronutrient malnutrition. Information on radiographic extent of disease was lacking, but results of bacillary density in sputum, another marker of disease severity were available and used in the multivariable analyses. In a recent study where adjustments were made for radiographic extent of disease, under-nutrition remained strongly associated with TB mortality [51]. HIV testing was offered but not accepted by all subjects, but Chhattisgarh is a low prevalence area for HIV infection with an estimated HIV prevalence of less than 0.3% of the adult population in 2009 [52]. The outcomes in the patients who defaulted could not be ascertained. We conducted a sensitivity analysis assuming a mortality rate of 15% in defaulters (documented in a recent cohort study)[53]. The results were similar to our original analysis, although the effect estimate was smaller (adjusted OR: 0.87 per unit increase in BMI; 95% CI: 0.78-0.97). Adjustments for socioeconomic status, smoking and alcohol consumption could not be made in the multivariable analysis. However nutritional status is strongly correlated with socio-economic status in India [12].

The strengths of this study are that it represents a large sample of rural patients studied over 6 years. The sampling of consecutive patients makes selection bias less likely, though because of the location and low-cost nature of services, the poor might have been over-represented in our cohort. The composition of patients reflects the status of patients diagnosed at both primary and secondary care level. The median heights in this study were identical to the median heights of rural men and women in a survey in 16 states in India [54]. This suggests that the levels of chronic under-nutrition in our patients were similar to other subjects in rural India, which enhances the generalizability of our findings to other rural populations in the country.

This study has some important implications. Under-nutrition was a highly prevalent co-morbidity associated with an increased risk of death in these patients. The under-nutrition in these patients was possibly a result of both active TB as well as pre-existing chronic under-nutrition. The evidence of pre-existing under-nutrition can be inferred from a variety of sources. 52% of patients had heights which fell below the 3rd percentile of the NCHS standards, indicating poor nutrition in childhood [31]. If under-nutrition was only disease related, patients with smear positive pulmonary TB (who have higher disease burden) would have had lower BMI and weights, and weights after successful treatment would have been normal. However BMI and weights were similar across categories of smear status in this study, and post-treatment weights continued to be low. Under-nutrition in adults in rural Chhattisgarh is highly prevalent. According to NFHS-3 figures,46% of women and 36% of men in rural Chhattisgarh were undernourished (BMI<18.5), while less than 3% of men and women were obese (>30.0 kg/m^2^)[12].

The finding of severe and life-threatening levels of under-nutrition in this cohort of patients from rural India raises the issue of its management. Neither current WHO guidelines for treatment of tuberculosis[55], nor the International Standards of Tuberculosis Care[56],discuss the importance of under-nutrition or nutritional support during treatment. The WHO guidelines on management of severe under-nutrition in adults however suggest hospitalization of such patients in view of their mortality risk, concomitant treatment of the medical illness, and dietary supplementation till a BMI of 18.5 is reached[57]. In this study following the WHO recommendations would have entailed hospitalization of nearly half of all diagnosed patients, a measure which was not feasible. 

We conclude that nutritional support should be considered for severely underweight patients with pulmonary TB to decrease their risk of mortality, although community based nutritional interventions for such patients in rural India, require further investigation. 
